# Efficacy of strength and aerobic exercise on patient-reported outcomes and structural changes in patients with knee osteoarthritis: study protocol for a randomized controlled trial

**DOI:** 10.1186/1471-2474-14-266

**Published:** 2013-09-12

**Authors:** Britt Elin Øiestad, Nina Østerås, Richard Frobell, Margreth Grotle, Helga Brøgger, May Arna Risberg

**Affiliations:** 1 Norwegian Research Center for Active Rehabilitation (NAR), Department of Orthopedics, Oslo University Hospital, Kirkeveien 166, 0407 Oslo, Norway; 2 National Resource Center for Rehabilitation in Rheumatology, Department of Rheumatology, Diakonhjemmet Hospital, Oslo, Norway; 3 Department of Orthopaedics, Lund University Hospital, Lund, Sweden; 4 Oslo University College, Oslo, Norway; 5 Department of Radiology, Oslo University Hospital Ullevaal, Oslo, Norway; 6 Department of Sport Medicine, Norwegian School of Sport Sciences, Oslo, Norway

**Keywords:** Knee, Osteoarthritis, Exercise therapy, Articular cartilage, Cost-effectiveness

## Abstract

**Background:**

Despite an extensive literature on treatment interventions for patients with knee osteoarthritis, studies comparing the efficacy of different exercise interventions and living the life as usual on quality of life, cartilage quality and cost-effectiveness are lacking. The aim of the present study is to compare the efficacy of two different exercise programs compared to a control group in individuals with established radiographic and symptomatic knee osteoarthritis on self-reported knee-related quality of life, knee pain, physical function, and cartilage quality.

**Methods/Design:**

A three-armed randomized controlled trial involving two exercise interventions and a control group of individuals doing as they usually do is described. The patients will have mild to moderate radiographic osteoarthritis according to the Kellgren and Lawrence classification (grade 2–3), and fulfill the American College of Rheumatology clinical criteria, be aged between 45 and 65 years, and have no other serious physical or mental illnesses. The patients will be randomly allocated to a strength exercise group; a cycling group, or a control group. The primary outcome is the Knee injury and Osteoarthritis Outcome Score knee-related quality of life subscale. Secondary outcomes include all five Knee Injury and Osteoarthritis Outcome Score subscales, morphological evaluation of cartilage including focal thickness, subchondral bone marrow edema, proteoglycan content and collagen degradation (measured using magnetic resonance imaging clinical sequences, T2 mapping and T1ρ), specific serum biomarkers, isokinetic muscle strength, maximal oxygen uptake, quality of life (EuroQol 5D), and self-efficacy (Arthritis Self-Efficacy Scale). A sample size calculation on the primary outcome showed that 207 individuals, 69 in each group, is needed to detect a clinically relevant difference of 10 points with 80% power and a significance level of 5%. Assessments will be conducted at baseline, 14 weeks, 1 year and 2 years post-randomization. The interventions will be a 14 weeks exercise program.

**Discussion:**

Although exercise therapy has been found to be effective in knee osteoarthritis, the knowledge of the underlying mechanisms for why exercise works is lacking. This study will contribute with knowledge on the efficacy of strength exercise versus cycling on patient-reported outcomes, cartilage quality and cost-effectiveness.

**Trial registration:**

Clinicaltrial.gov Identifier: NCT01682980.

## Background

Recent recommendations for treatment of knee osteoarthritis suggest exercise and physical activity in combination with patient education as first line treatments to reduce pain and improve function [1-6]. During the last decades, several randomized controlled trials (RCTs) summarized in systematic reviews and meta-analyses show that exercise and physical activity are important interventions in a rehabilitation phase as well as in daily life in reducing pain and improving function [7,8]. New evidence support that exercise in almost any types works; aquatic exercises [9] or land-based exercises [10], aerobic exercises [11] or strength exercises [12-14], high-intensity strength exercise or low intensity exercises [15,16]. Nevertheless, the effect sizes are low to moderate. Studies have shown similar effect sizes for exercise therapy and pain reliving drugs for patients with knee osteoarthritis, but exercise therapy have fewer side-effects than may be seen for drug therapy [3].

Despite of a huge amount of literature in this field, we need more high-quality RCTs that give new knowledge with respect to the efficacy of different types of exercise and the optimal exercise intensity and frequency [2,6,8,17,18]. Adherence to exercise is another important aspect associated to the efficacy of exercise in patients with knee osteoarthritis. Wang et al. [12] showed a possible association between high adherence to exercise and improved pain and function. We currently know too little about the dose–response relationship in exercise interventions on patients with osteoarthritis [12]. All existing literature concerning effective treatment methods for osteoarthritis inquire clearer guidelines on exercise frequency, duration, and intensity. Furthermore, studies identifying those patients responding well to exercise (responders) and those who do not (non-responders), are lacking. In addition, there is little knowledge on which subgroups benefit more (or less) from exercise interventions. For instance, age, obesity, radiographic severity, previously knee injury, and level of physical activity are factors that we need more specific knowledge about [19].

RCTs evaluating the efficacy of strength training versus aerobic exercise on knee-related quality of life (QOL), pain and physical function in patients with mild to moderate knee osteoarthritis are lacking [6,17]. Strength deficits may be caused by several aspects of muscle function such as atrophy, pain inhibition, or activation failure [20]. Thus, both neuromuscular exercises and strength exercises should be included in a strength program to increase muscle function [20,21]. Few studies have evaluated the effect of neuromuscular exercises targeting hip control in combination with progressive strength exercises in patients with knee osteoarthritis [22]. Different types of exercises such as strength training including neuromuscular exercises versus aerobic exercise might induce different adaptations in patients with knee osteoarthritis. Hence, an investigation on how various training methods might influence knee-related QOL, pain, and physical function is necessary. To ensure a reliable evaluation of the efficacy of an intervention, a control group that is not receiving a particular intervention should be included in RCTs.

Sparse evidence exists with respect to the efficacy of different exercise interventions on structural progression of the disease [23]. Significant biomarkers of structural and compositional changes to bone, cartilage, and synovium may be identified using magnetic resonance imaging (MRI). With the understanding that structural changes in the knee joint starts early and that the osteoarthritis disease process affects several joint structures, MRI has now been recommended to assess cartilage and other knee joint pathologies in clinical trials. Quantitative MRI techniques can capture tissue morphology and biochemical composition [24]*.* Monitoring changes in collagen integrity and proteoglycan content may give us valuable insight into the understanding of mechanisms explaining why exercise therapy reduces knee pain.

Osteoarthritis seems to be both a mechanically and an inflammatory driven disease [25]. Thus, biomarkers from proteins in serum and synovium have recently been used to study changes in the knee osteoarthritis disease process. Furthermore, some recent studies have reported the effect of exercise interventions on circulating cartilage markers [26,27]. Exercises have shown to cause rise in circulating cartilage markers like cartilage oligomeric matrix protein (COMP), aggrecan and C-terminal telopeptides of collagen type II (CTX-II) [27,28]. Additionally, exercises have shown to increase the production of the anti-inflammatory mediator interleukin-10 [29].

The economic burden of knee osteoarthritis is growing with respect to surgical interventions, non-pharmacological and pharmacological treatments, and consequently social indirect costs. However, according to a systematic review by Pinto et al. [30] there is limited evidence for the cost-effectiveness of non-surgical treatments for the management of knee osteoarthritis. Thus, cost-effectiveness analyses comparing conservative treatment with usual care are needed.

### Aims

In patients with mild to moderate knee osteoarthritis, the purpose of this study is to evaluate the efficacy of a strength exercise program versus an aerobic exercise program compared to a control group doing as they usually do using knee-related QOL, physical function and structural progression of the disease (cartilage quality). Furthermore, we aim to do cost-effectiveness analyses on exercise interventions compared to control group. One group will have tailored strength exercises, a second group will perform cycling, and a third control group will do as they usually do (but are asked to not start physiotherapy treatment or exercises the first 4 month of the study period). The primary outcome will be knee-related QOL at the 1 year follow-up. Secondary outcomes include pain, other symptoms, function in activities of daily living (ADL) and sport and recreation, thigh muscle strength, aerobic capacity, cartilage morphology, and biochemical composition as well as conventional radiographic evaluation of the knee joints. In addition, an explorative analysis of prognostic factors for the main outcome after 2 years will be carried out. The intervention will start with a 2 week preparation phase followed by 12 weeks intervention phase. Assessments will be performed before the intervention starts (baseline), after 14 weeks, 1 year, and 2 years post-randomization. The study is a superiority trial with the following hypotheses:

1. Clinical outcomes

In patients with mild to moderate knee osteoarthritis:

• Strength exercise and cycling are both more effective than doing as usual in improving knee-related QOL during a 1 year follow-up.

• Strength training and cycling are both more effective than doing as usual in improving knee function during a 1 year follow-up.

• Strength training and cycling are both more effective than doing as usual in postponing joint replacement at 2 and 5 years post-randomization.

• Strength exercise is more effective than cycling in improving knee function.

2. Structural outcomes

In patients with mild to moderate knee osteoarthritis:

• Both strength exercise and cycling interventions will have superior effects on cartilage morphology, biochemical composition, and radiographic joint space compared to the control group.

• Cycling intervention is superior to strength exercise in preserving cartilage morphology and biochemical composition.

3. Cost-effectiveness

• Strength exercise and cycling are both more cost-effective than doing as usual in patients with mild to moderate knee osteoarthritis.

• A change in the Knee injury and Osteoarthritis Outcome Score (KOOS) subscales of 8–10 points are clinically important differences in patients with mild to moderate knee osteoarthritis.

4. Subgroup analysis

• Those who respond well to the exercise programs on KOOS pain have higher quadriceps strength and maximal oxygen consumption at baseline (reflecting higher physical activity level) than those who do not respond to strength training and cycling.

• Patients with mild radiographic osteoarthritis at baseline will respond significantly better to the interventions compared to those with moderate radiographic osteoarthritis at baseline on KOOS pain and QOL during the 2 year follow-up.

• Patients classified as “non-progressors” of structural changes will respond significantly better to the exercise intervention compared to those who are defined as “progressors” over a period of 2 years (progressors defined as Prasad et al. [31]).

## Methods/Design

This study presents a RCT adhering to the SPIRIT 2013 statement [32] including three groups: two intervention groups (strength exercises and cycling) and one control group (doing as usual, but those in the control group are told to not start physiotherapy treatment during the first 4 months of the study period). Outcome assessments will be performed at baseline, after the intervention (14 weeks), 1 year after baseline, and 2 years after baseline. In addition, those who refuse to take part in the RCT will be asked to participate in an inception cohort including baseline assessments and a 2 year follow-up. Data from these patients will be used together with the data from the RCT in the analyses of prognostic factors for severe knee osteoarthritis.

### Recruitment

**Figure 1:**
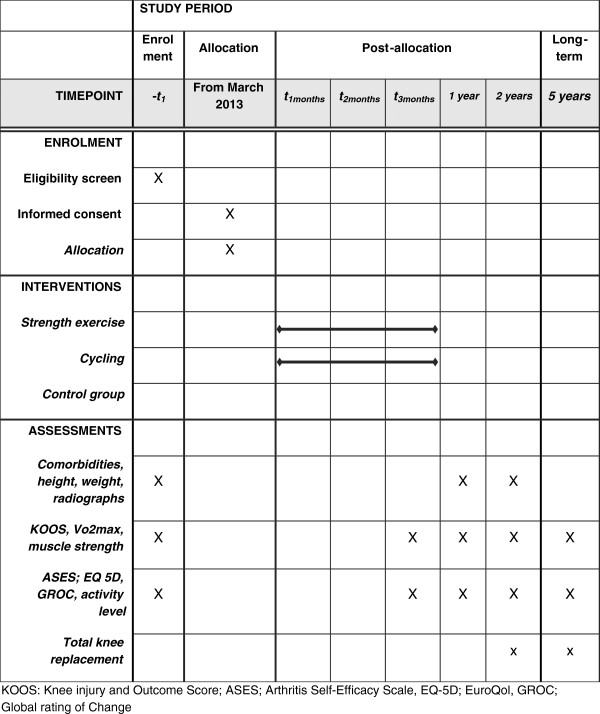
The schedule of enrolment, interventions, and assessments.

The subjects will be included from the primary health care in Oslo, and at university hospitals in Oslo and Akershus (Figure 1). A part of the study participants will be included from the Musculoskeletal Pain in Ullensaker Study (MUST Part III) [33]. There will be one screening of patients already participating in the MUST and one screening of patients from the waiting lists at the university hospitals and other hospitals in the Oslo area.

MUST Part I is a population based postal survey that is sent to all inhabitants between 40 and 79 years old in Ullensaker Municipality (n = 12 000). Those who self-report osteoarthritis in their hands, knees or hips, are invited to a clinical examination (MUST Part II) at Diakonhjemmet hospital (estimated n = 600). Among these, persons with knee osteoarthritis in line with the inclusion and exclusion criteria will be recruited to this RCT (MUST Part III) [33].

### Inclusion criteria

• Women and men aged 45–65 years

• Clinical knee osteoarthritis according to the American College of Rheumatology Clinical Criteria [34]

• Kellgren and Lawrence (KL) radiographic osteoarthritis grade 2 and 3 (mild to moderate radiographic osteoarthritis)

### Exclusion criteria

• Severe knee osteoarthritis according to the KL classification (grade 4)

• Other known major musculoskeletal impairments in the lower extremities or the back or prostheses in any joint of the lower extremities

• Known serious coronary heart diseases or cancer

• Body mass index >35

• Scheduled for surgery in any joint

• Known mental or psychological diseases

• Known drug abuse

• Persons who already perform sports related moderate physical activity more than two times a week

• Contraindications for MRI

• Not speaking Norwegian language

### Randomization and blinding

The participants who agree to participate in the RCT will be randomly allocated into one of three groups: the strength exercise group; the cycling group, or the control group. A computer-generated randomization schedule will be used to generate the randomization lists using blocks of six. A research coordinator not involved in the randomization will prepare the envelopes for the randomization procedure. The envelopes will be opened by the patients after the baseline test. The study will be single-blinded such that the assessors will be blinded to group allocation.

### Primary outcome

• KOOS knee-related QOL

### Secondary outcomes

• KOOS pain, other symptoms, activities of daily living (ADL), and function in sport/recreation

• Health-related quality of life (EuroQOL-5D, EQ-5D)

• Radiographic knee osteoarthritis progression (joint space and KL score)

• Isokinetic muscle strength

### Exploratory outcomes

• Cartilage morphology measures (MRI–T2 mapping and T1ρ)

• Serum proteins

• Maximal oxygen consumption (VO_2max_)

• Self-efficacy for pain (Arthritis Self-Efficacy Scale, ASES)

### Assessments

#### Primary outcome

##### Knee-related QOL

The KOOS [35] will be included to measure knee-related QOL at baseline, after the intervention, and after 1 year and 2 years post-randomization. The KOOS is a self-administered knee-specific questionnaire containing 5-item Likert scales on pain, other symptoms, ADL, function in sports and recreation and knee-related QOL. KOOS has been validated for subjects with post-traumatic osteoarthritis [36] and includes The Western Ontario and McMaster Universities Arthritis Index (WOMAC), which is widely used to measure pain and physical function in patients with osteoarthritis [37]. Primary outcome will be the subscale on knee-related QOL of life as this subscale is sensitive for detecting impaired function for patients with knee osteoarthritis [38]. All the KOOS subscales will be included separately as secondary outcomes.

#### Secondary outcomes

##### Radiological examination

Conventional radiographic procedure will be performed at baseline and at the 2 year follow-up. For the frontal plane images, the SynaFlexer® frame for standardized positioning as described by Kothari et al. [39] will be used. In this protocol, a 10° caudal beam angulation ensures alignment of the beam with the medial tibial plateau. A standardized degree of knee flexion (20°) and external foot rotation (10°) are achieved using the frame. X-rays will also be taken in a lateral view and tangentially of the patella, bilaterally. The KL classification system [40] will be used for grading the radiographs (Table 1).

**Table 1:** **Kellgren and Lawrence classification system**[40]

**Grade**	**Description**
0	No changes
1	Doubtful narrowing of joint space and possible osteophytic lipping
2	Definite osteophytes and possible narrowing of joint space
3	Moderate multiple osteophytes, definite narrowing of joint space, and some sclerosis, and possible deformity of bone ends
4	Large osteophytes, marked narrowing of joint space, severe sclerosis and definite deformity of bone ends

Grade 2 will be modified according to suggestions by Felson et al. [41] such that definite osteophytes will be registered as grade 2a and definite osteophytes and possible joint space narrowing will be registered as 2b. In retrospect, all the radiographs will be reclassified blindly and independently by an experienced radiologist, according to the above defined criteria. Minimal joint space width (JSW) will be measured and used to define progression. Using a semi-flexed view and at least 2 years follow-up time, JSW measurements have been shown to be sensitive to change [42] and have shown good evidence for reliability and responsiveness [43]. A standardized response mean (SRM) of 0.71 (95% CI of 0.44-0.98) has been calculated for conventional radiographic JSW in knee osteoarthritis trials using semi-flexed views with a trial duration of more than 2 years [42]. In order to evaluate structural progression using changes in JSW, a reliability study will be performed to establish the smallest detectable difference (SDD) within this study for the JSW measurements. This is based on the OARSI-OMERACT recommendations for structural progression using conventional radiographic measurements. The SDD will be determined from Bland and Altman plots, meaning a change in JSW beyond the measurement error. Progression and cut-off for radiological progression will be defined based on the measurement error within this study.

##### Health related QOL

Health-related QOL will be assessed by the EQ-5D (EuroQoL Group 1990) [44].

The cost-effectiveness analysis will compare the potential effect of the interventions by using the KOOS QOL and the EQ5D as the measure of effectiveness. Costs of the study treatment (direct costs) will be estimated using a bottom-up approach. We will register the number of exercise sessions, personnel involved and their time spent, and materials and practice space used. Costs to the healthcare system incurred due to knee osteoarthritis (indirect costs) will be recorded by a cost diary, including registrations of number of visits to a general practitioner, physical or manual therapist, medical specialist, social worker, and alternative therapist, number of days of hospitalization and/or rehabilitation, use of medication (both on prescription and over the counter medication), and number of days of sick leave from work. The costs of work absenteeism will be estimated by the number of days absent from work multiplied by the average wage rate. The cost diary will be recorded at baseline and after 3, 6, 9, and 12 months post-randomization by calling the included study participants at 6 and 9 months.

##### Muscle strength tests

Isokinetic equipment (Biodex 2000) will be used to evaluate the mm. quadriceps and hamstrings muscle performance. Isokinetic muscle testing is commonly used in the literature and has been validated for measurement of muscle strength performance and the reliability has been reported to be adequate [45]. A procedure involving concentric knee extension and knee flexion in a range of 90 degrees with 5 repetitions at 60°/sec will be performed.

#### Explanatory outcomes

MR imaging and collection of blood samples will commence when 36 individuals have been included in each group (i.e. performed on the last 33 individuals of each group) due to the high costs involved. MR images will be obtained bilaterally (i.e. both knees) and blood samples will be drawn at baseline, post-intervention, and at 1 and 2 years post-randomization.

##### Magnetic resonance imaging

MRI examinations will be conducted using a 3.0 Tesla Philips INGENIA scanner (Release 4.1.2.1, from August 2012) with 8-channel knee-coil at the Department of Radiology at Oslo University Hospital. MRI has shown to have inherent strength and advantages to visualize tissue pathology and has shown good reliability and responsiveness [46]. A separate MRI method protocol will be developed to assess the utility, limitations, validity and reliability of T2 mapping and T1ρ sequence methods in evaluating the quality of cartilage in patients with mild knee osteoarthritis. The MRI evaluation will include morphological evaluation of cartilage, detecting focal thickness reduction or defects. Focal areas of subchondral bone marrow edema will be noted. Assessment of cartilage quality (proteoglycan content and collagen degradation) will be made using two sequences: T2 mapping and T1ρ. T2 mapping have been shown to correlate with collagen degeneration in patients with knee osteoarthritis [47] whereas T1ρ has been shown to detect changes in the proteoglycan content of the cartilage. Both were suggested as viable tools for detecting early degeneration and progression of osteoarthritis disease and both have shown biochemical changes in the knee articular cartilage in the absence of radiographic evidence of knee osteoarthritis [48].

##### Laboratory measures

Molecular biomarkers of cartilage turnover will be assessed in serum. Serum samples of venous blood will be collected at the same visit as image acquisition (3 vacutainer tubes, each containing 6 ml). After being centrifuged at 1800 g for 10 minutes, samples will be stored at −80 degrees Celsius in 1 mL aliquot tubes. We will investigate the longitudinal change of cartilage specific biomarkers (including, but not limited to a specific fragment of Aggrecan (ARGS), Collagen −9 and Cartilage Oligomeric Matrix Protein (COMP) and cytokines (including, but not limited to IL-6, TNF-α and TGF-β).

##### Maximal oxygen consumption

Maximal oxygen consumption (VO_2max_) will be measured on an ergometer cycle (Monark 839E, Sweden) at baseline, at post-intervention, and at the 1 and the 2 year follow-ups. A cardiovascular examination will be performed at the screening to exclude those with serious and unstable coronary heart diseases. The test protocol includes a 20 minute progressive warm-up at 45–90% VO_2max_, followed by an all-out incremental “ramp” test, lasting approximately 4–6 minutes. During the “ramp” test, the cadence will remain as steady as possible at 90 repetitions per maximum, and the workload will increase by 25 Watts every 30 second, to a supramaximal workload and totally exhaustion (rate of perceived exertion 17–19).

##### Self-efficacy for pain

Several studies including patients with osteoarthritis have found that a patient’s perceived self-efficacy is related to health outcomes and the course of disease progression [49,50]. ASES will be included as a measure of the patients’ self-efficacy for pain. Perceived self-efficacy is defined as a person’s judgment or belief of their ability to change, manage or execute tasks related to pain [50]. It is concerned not with the skill one has, but measures a changeable psychological aspect of pain [51]. A modified version of ASES contains 11 questions regarding the patient’s certainty to perform various tasks related to pain and symptoms, where each item is rated from 1 (very uncertain) to 5 (very certain). Good concurrent validity and internal reliability has been reported for the original version of the scale [51].

##### Other assessments

Background variables such as height, weight, physical activity, type of work or disability pensions, range of motion, knee joint alignment measured with an inclinometer [52], numeric change scale, analgesics consumption or other medications, previously knee injuries, and adverse events will be collected at the baseline test, the post-intervention test and at 1 and 2 year follow-ups. Cases of joint replacement will be recorded at the 2 year follow-up.

### Interventions

The interventions will be delivered by guided physiotherapists at selected physical therapy institutes in the Oslo and Akershus area. The strength exercise program will be based on previously developed exercise programs [14], focusing on heavy resistance training and neuromuscular training including one leg exercises and balance training. The program has been planned according to American College of Sports Medicines (ACSMs) guidelines for strength progression in healthy adults [53]. The aerobic exercise will be performed to improve physical function and cartilage quality and the dosage has been planned according to general guidelines for training parameters in people with pain associated to osteoarthritis, as developed by the American Geriatrics Society [2]. The intervention programs will last for 12 weeks with 2–3 training sessions per week. In addition, the patients will go through a pre-phase of 2 weeks to prepare for the intervention programs. The patients in all three groups will be told to stick to the exercise program or usual care for the 12 weeks of intervention to make sure they do not increase or change usual activity level parallel with the intervention. The included patients will be followed closely to ensure that the exercises are performed adequately and to ensure the progression of intensity. A description of the interventions and the response to the program depending on patient characteristics will be performed in a separate study. The definition of responders will be defined according to the OMERACT-OARSI set of responder criteria, including improvement in at least two of the following three criteria: pain ≥ 20% and absolute change ≥ 10%, or physical function ≥ 20% and absolute change ≥ 10%, or patient’s global assessment ≥ 20% and absolute change ≥ 10% [54]. A brief description of the three interventions can be seen below:

#### Strength exercise intervention

The strength exercise program will be delivered 2–3 times per week for 12 weeks, 8–10 repetitions maximum (RM) in 3 series with approximately 30 seconds to 1 minute pause between the series. One of the training sessions may be performed at home. The patients will go through a 2 weeks phase before the strength program including neuromuscular exercises and strength exercises on low intensity. The patients will warm up 5–10 minutes on an ergometer cycle or a treadmill according to the patients’ desire. The following muscle groups will be trained: Quadriceps and hamstrings, hip abductors and extensors, and calf muscles. A home exercise program will be delivered including one leg exercises and balance exercises. Progression will follow a 2+ principle [55]. For instance, when the study participant is able to perform 2 more repetitions in a set of 3×8, more loads are required.

#### Aerobic exercise intervention

In the present study, the main aim of the aerobic exercise is to improve cartilage quality, in addition to the general health effects physical activity gives. Both overloading and underloading may cause cartilage degradation, but moderate loading has been shown to be beneficial for joint health because of mechanosensitive chondroprotective pathways [23,56]. However, these pathways need to be further elucidated to identify the causation. Based on the moderate loading benefits, ergometer cycling for 45 minutes 2–3 times a week, including 10 minutes warm up, 30 minutes on moderate loading (75% of max heart rate) and 5 minutes cool down will be required. For instance, a patient with a maximal heart rate of 160 and rest heart rate of 60 will be required to cycle at a heart rate of about 135 using the formula for heart rate reserve (160-rest heart rate of 60 × 0.75 + rest heart rate of 60). The patients who will be allocated to the cycling group will be told to measure their rest heart rate before the intervention starts to ensure moderate loading during the cycling sessions.

#### Compliance to the interventions

The patients will be asked to report all training sessions in detailed training diaries distributed to the patients after randomization, including type of exercise, loading, and progression. Pain during training will be reported using the visual analogue scale (0–10) (VAS). A cut off value of 4–6 is suggested to be acceptable pain during or after the exercise session in patients with knee osteoarthritis [57]. The diaries will be controlled weekly by the physiotherapist to ensure compliance with the program. An optimal goal is that the subjects should complete minimum 80% of the planned exercise sessions according to ACSMs guidelines.

### Control group

A control group doing as usual will be included to control for the natural course of knee osteoarthritis. They will be told to not start a new physiotherapy treatment period the first 4 months after inclusion in the study. The group will be tested at baseline, after 14 weeks, after 1 year and 2 years with the same tests as for the intervention groups. Those who are in the control group will get exercise advice according to our intervention programs after they have finished the first 4 months of the study if they are interested.

### Sample size calculation

Primary outcome for the RCT will be the KOOS subscale knee-related QOL (0–100 scale). A clinical important difference in the KOOS subscales has been suggested to be 8–10 points [58]. Based on 10 points expected difference between the intervention groups and the control group and a standard deviation of 20 on QOL, 63 patients are needed in each group with significance level of 0.05, and power of 80% using Popocks formula: n = 2σ^2^/(μ_1_- μ_2_)^2^ * f(α,β). With an estimated drop-out rate of 10% a total number of 207 subjects will be randomized either into the strength exercise group (n = 69); the cycling group (n = 69), or the control group (n = 69).

### Statistical methods

The analyses will be performed using intention-to-treat mixed linear models and Student t-tests. For mixed models, the baseline score will be included as a covariate, the subjects as random effect and the treatment condition as fixed factor. We will consider using the closest match missing data imputation for repeated measures data [59]. The cost-effectiveness analysis will be conducted from the societal perspective and according to the intention-to-treat principle. An incremental cost-effectiveness ratio will be calculated by dividing the between-group difference in costs by the between-group difference in effects. Cost-effectiveness ratios will be estimated using bootstrapping techniques and graphically presented on cost-effectiveness planes. Acceptability curves and net monetary benefit will also be estimated. Sensitivity analysis on the most important cost drivers will be performed in order to assess the robustness of the results. The statistical analyses will be discussed and co-worked with our statisticians at the Oslo University Hospital and the Norwegian School of Sport Sciences, respectively, and with our international collaboration groups.

### Ethical perspectives and project funding

The study is approved by the Regional Ethical Committee and The Data Inspectorate in Norway (Ref. 2012/334). All the included subjects will sign an informed written consent, and will be able to withdraw from participation in the study at any time point. The Research Council of Norway is funding the first part of the project including the 1 year follow-up in a three years full time post doc position (Ref. H10/213335). Furthermore, the study will be collaborated between researchers from the Oslo University Hospital (Department of Orthopeadics and Department of Radiology), Diakonhjemmet Hospital, Oslo University College, Akershus University Hospital (Department of Orthopaedics) and Lund University.

## Discussion

Guidelines and meta-analyses request more RCTs evaluating type, intensity, and frequency of exercise which may contribute in the knowledge of dose–response relationship. Furthermore, we do not know underlying mechanisms for why exercise work analyzing data from imaging or blood samples. We have presented the rationale and design of a RCT for investigating the efficacy of different types of exercise interventions on patient-reported clinical outcomes as well as cartilage quality and cost-effectiveness in patients with mild to moderate knee osteoarthritis. We argue that this study will contribute with important knowledge that is requested. If the study succeeds in demonstrating a significant positive effect on clinical, radiological, and cost-effectiveness outcomes, the potential gain is large because of the increasing number of patients suffering from knee osteoarthritis in the society.

### Conclusion

This study protocol presents a RCT on the efficacy of different types of exercise interventions on patient-reported clinical outcomes as well as cartilage quality and cost-effectiveness in patients with mild to moderate knee osteoarthritis. The RCT will contribute with knowledge on the efficacy of strength exercise versus cycling on patient-reported outcomes, cartilage quality and cost-effectiveness.

## Competing interests

The authors declare no competing interests.

## Authors’ contributions

BEØ (project leader)(britt.e.oiestad@nimi.no): has written the protocol, will screen and include patients, be one of the blinded testers, perform data analysis and be the main author of articles on the primary aim of the study. NØ (nina.osteras@medisin.uio.no): contributed to ideas in the protocol, will analyze data and publish articles. RF (Richard.frobell@med.lu.se): contributed to ideas on the overall protocol and in particular on the magnetic resonance imaging (MRI) protocol, and will publish articles on MRI data. MG (margreth.grotle@medisin.uio.no): contributed to ideas on the overall protocol, responsible for the cost-effectiveness analyses, and will publish articles accordingly. HB (helga@brogger.no): responsible for reading the MRI images, and will evaluate the validity and reliability of the T2 mapping and T1ρ sequences in a separate MRI project. MAR (m.a.risberg@nih.no): contributed with ideas on the overall protocol, responsible for the MRI protocol, will analyze data and publish articles on cartilage health related to exercise interventions. All authors read and approved the final manuscript.

## Pre-publication history

The pre-publication history for this paper can be accessed here:

http://www.biomedcentral.com/1471-2474/14/266/prepub
